# TriSwinUNETR lobe segmentation model for computing DIR-free CT-ventilation

**DOI:** 10.3389/fonc.2025.1475133

**Published:** 2025-02-17

**Authors:** Gabriela Roque Oliveira Nomura, Aaron T. Luong, Ananya Prakash, Annabelle Alemand, Tanish Bhowmick, Alisa Ali, Jaimie Ren, Basil Rehani, Girish Nair, Richard Castillo, Yevgeniy Vinogradskiy, Edward Castillo

**Affiliations:** ^1^ Department of Biomedical Engineering, The University of Texas at Austin, Austin, TX, United States; ^2^ Division of Pulmonary and Critical Care, William Beaumont University Hospital, Royal Oak, MI, United States; ^3^ Department of Radiation Oncology, Emory University School of Medicine, Atlanta, GA, United States; ^4^ Department of Radiation Oncology, Thomas Jefferson University, Philadelphia, PA, United States

**Keywords:** lobe segmentation, CT-ventilation, artificial intelligence, transformer networks, functional radiotherapy, deformable image registration, medical image segmentation

## Abstract

**Purpose:**

Functional radiotherapy avoids the delivery of high-radiation dosages to high-ventilated lung areas. Methods to determine CT-ventilation imaging (CTVI) typically rely on deformable image registration (DIR) to calculate volume changes within inhale/exhale CT image pairs. Since DIR is a non-trivial task that can bias CTVI, we hypothesize that lung volume changes needed to calculate CTVI can be computed from AI-driven lobe segmentations in inhale/exhale phases, without DIR. We utilize a novel lobe segmentation pipeline (TriSwinUNETR), and the resulting inhale/exhale lobe volumes are used to calculate CTVI.

**Methods:**

Our pipeline involves three SwinUNETR networks, each trained on 6,501 CT image pairs from the COPDGene study. An initial network provides right/left lung segmentations used to define bounding boxes for each lung. Bounding boxes are resized to focus on lung volumes and then lobes are segmented with dedicated right and left SwinUNETR networks. Fine-tuning was conducted on CTs from 11 patients treated with radiotherapy for non-small cell lung cancer. Five-fold cross-validation was then performed on 51 LUNA16 cases with manually delineated ground truth. Breathing-induced volume change was calculated for each lobe using AI-defined lobe volumes from inhale/exhale phases, without DIR. Resulting lobar CTVI values were validated with 4DCT and positron emission tomography (PET)-Galligas ventilation imaging for 19 lung cancer patients. Spatial Spearman correlation between TriSwinUNETR lobe ventilation and ground-truth PET-Galligas ventilation was calculated for each patient.

**Results:**

TriSwinUNETR achieved a state-of-the-art mean Dice score of 93.72% (RUL: 93.49%, RML: 85.78%, RLL: 95.65%, LUL: 97.12%, LLL: 96.58%), outperforming best-reported accuracy of 92.81% for the lobe segmentation task. CTVI calculations yielded a median Spearman correlation coefficient of 0.9 across 19 cases, with 13 cases exhibiting correlations of at least 0.5, indicating strong agreement with PET-Galligas ventilation.

**Conclusion:**

Our TriSwinUNETR pipeline demonstrated superior performance in the lobe segmentation task, while our segmentation-based CTVI exhibited strong agreement with PET-Galligas ventilation. Moreover, as our approach leverages deep-learning for segmentation, it provides interpretable ventilation results and facilitates quality assurance, thereby reducing reliance on DIR.

## 1 Introduction

The lungs are situated within the thoracic cavity, enclosed by the ribcage. Although the right and left lungs appear similar, they exhibit notable anatomical differences. The right lung consists of three lobes: right upper lobe (RUL), right middle lobe (RML), and right lower lobe (RLL). The left lung consists of only two: left upper lobe (LUL) and left lower lobe (LLL). Lobar boundaries are characterized by fissures that appear as thin white lines on computed tomography (CT) scans. There is variability in lobe fissures, which may be complete when the lobes are connected solely at the hilum by the bronchi and pulmonary vessels, incomplete when there are areas of parenchymal fusion between the lobes, or entirely absent (1). In the right lung, the oblique fissure divides the lower and middle lobes, and the horizontal fissure divides the upper and middle lobes (2). The left lobe contains only one oblique fissure separating the upper and lower lobes (2). Radiologists and machine learning models alike identify the fissure locations in the lungs to determine the boundaries between lobes and subsequently perform the task of lobe segmentation.

Lobe segmentation is crucial for various medical applications, including disease diagnosis, severity assessment, and treatment planning (3). Previous research has shown that lung regions receiving high doses of radiation (> 20 Gy) experience a decrease in post-treatment lung function, as measured by decreased CTVI values (4). To improve lung cancer treatment, functional radiotherapy has been proposed. This approach aims to avoid delivering high radiation doses to high-functioning, or high-ventilated, lung areas during radiotherapy treatment planning. Methods to calculate CTVI typically use deformable image registration (DIR), in which lung voxels are registered from the inhale to the exhale phase of the breathing cycle, and the displacement of each voxel is measured (4). However, iterative DIR is a non-trivial task that can potentially bias CTVI due to its long computation time, potential inaccuracies in alignment, and inherent uncertainty (5). Therefore, the use of a DIR-free automated lung lobe segmentation method as a means for calculating CTVI based on volume changes is a potential avenue to be explored.

While healthy patients pose little challenge to existing lobe segmentation methods, the accuracy of the segmentations can be significantly worsened by the presence of disease states. It has been demonstrated that conditions such as COVID-19 can affect the appearance of lung tissue and cause existing methods to fail (6). Similarly, parenchymal fibrosis associated with chronic obstructive pulmonary disorder (COPD) can pose similar challenges to existing methods since the density of the fibrotic tissue is similar to that of tissue outside the lung, which can obscure fissures and affect thresholding-based algorithms (6).

**Figure 1:**
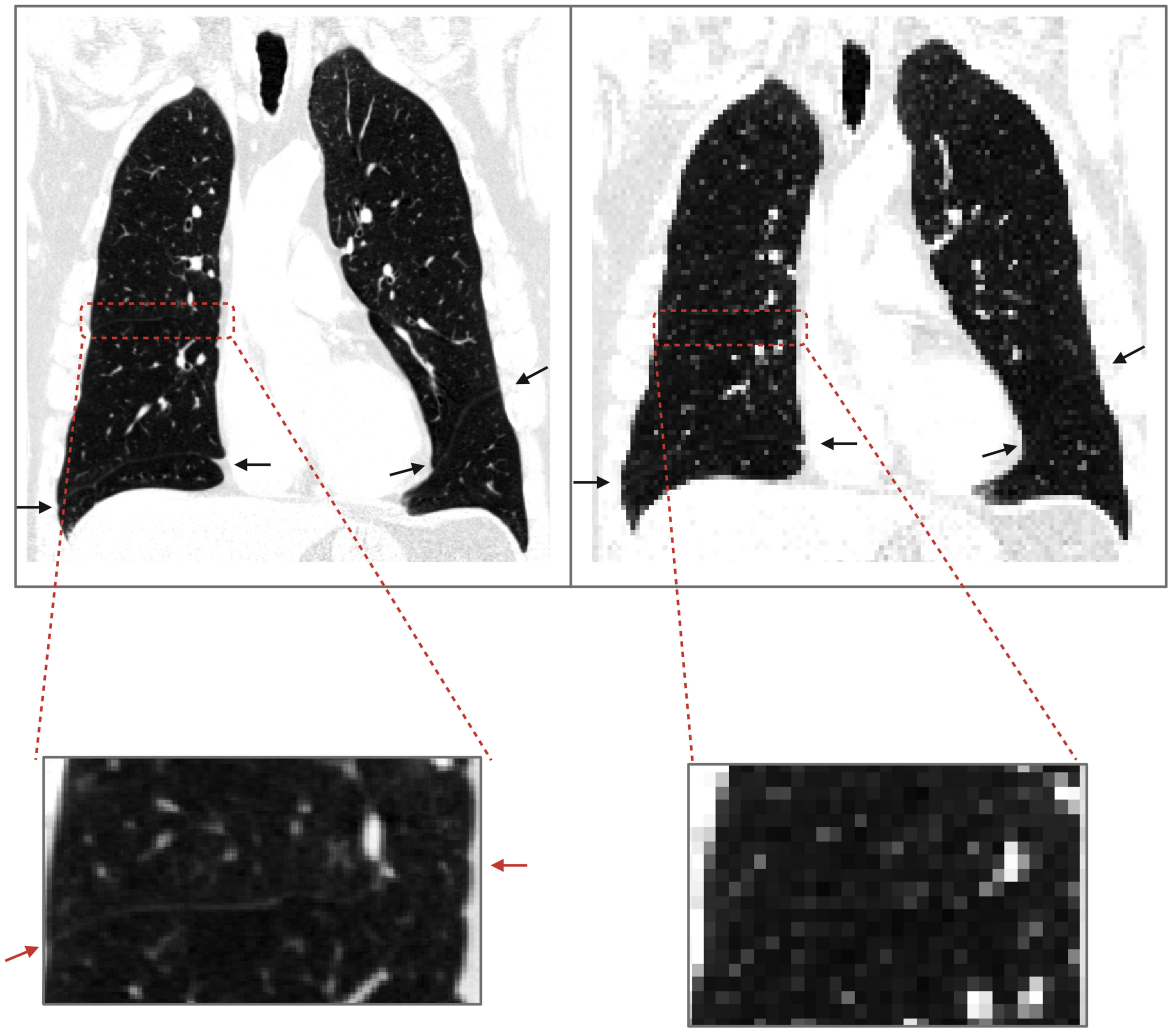
Loss of contextual information upon downsampling. The image on the left is a COPD Gold 1 case at resolution 512x512x649; the endpoints of fissures are shown with arrows. On the right, the same image was downsampled with nearest neighbor interpolation to 128x128x128, and upon visual inspection, the horizontal fissure on the right lung is not clearly identified.

Previous works on deep learning-based segmentation have been faced with the issue of downsampling images without losing substantial contextual information in order to fit the memory capacity of graphics processing units (GPUs) needed for model training. As shown in **Figure 1**, fissure identification is challenging at lower resolutions, but due to memory requirements, it is a common procedure to downsample original CT scans from a full-resolution to a lower-resolution space. To preserve contextual information, previous works have employed a random sampling tactic, resizing the original 512x512x256 CT scan to a lower resolution of 256x256x128 and then random sampling in 128x128x64 patches (7). The issue with such approaches, however, is in the first downsampling step, which diminishes the resolution of the images by a factor of 2, thus possibly leading to a loss of essential information that could have guided the model to a better result.

While convolutional neural networks (CNNs), proposed in the 1980s, have been widely used for medical imaging segmentation tasks, newer machine learning models have improved upon the basic CNN. Ronneberger et al. took the basic CNN structure to create the U-NET, widely used for medical imaging segmentation tasks. This “U-shaped” network consists of an encoder, a contracting path to capture contextual information, and a decoder, an expanding path that enables localization (8). In contrast to CNNs which are notably constrained to the local features captured by the kernel, the innovative transformer architecture provides the capability to capture more global features. Recent advancements in large language models are in large part thanks to the transformer architecture, introduced in the famous “Attention is All You Need” paper (9). Expanding upon the basic transformer network, the vision transformer (ViT) provided the precedent and foundation for implementing transformers in computer vision tasks (10). The UNETR architecture developed by Hatamizadeh et al. replaces the convolutional encoding arm of a traditional UNET with a ViT encoder, achieving state-of-the-art performance at the time in the Beyond the Cranial Vault (BTCV) abdominal CT multi-organ segmentation challenge (11). In order to translate images into a format compatible with the ViT, 16x16 pixel large patches of the image are taken and linearly projected. However, images often vary in scale and require higher resolution than the 16x16 patches can capture. The Swin Transformer resolves this limitation by dividing the image into a variety of patches ranging from 4x4 to 16x16 in order to capture the different scales and resolution of details in an image (12). This approach generalizes into the third dimension and is utilized in the SwinUNETR, replacing the ViT encoder with a Swin Transformer. This improved architecture is demonstrated to provide superior performance compared to its ViT-based predecessor in the BTCV challenge (13).

In this study, we propose the novel three SwinUNETR (TriSwinUNETR) ensemble pipeline network for CT lobe segmentation, using the proven state-of-the-art transformer-based machine learning architecture. Furthermore, we break down the lobe segmentation task into three steps to prevent loss of contextual information caused by image downsampling. Then, we train the machine learning pipeline in multiple datasets and disease states to ensure that the model is generalizable to multiple patients. Lastly, we test the model on a clinical task for calculating CTVI of lung cancer patients prior to undergoing radiotherapy.

## 2 Materials and methods

### 2.1 COPDGene and preprocessing

CT images from the COPDGene dataset were used to train the three SwinUNETR networks comprising our TriSwinUNETR model. COPDGene data was acquired from an observational study conducted to identify genetic factors that contributed to COPD (14). Images were acquired using multi-detector CT scanners, with 3D volumetric scans acquired on both full inspiration (200 mAs) and end-of-normal expiration (50 mAs) (14). For each patient, the scan is composed of sub-millimeter spaced (0.625 - 0.9 mm) 512x512 slices with a pixel spacing of 0.5 mm (14). The severity of COPD is categorized by a value for the Global Initiative for Obstructive Lung Disease, or GOLD score. GOLD scores range from 0 to 4, where an increasing score denotes increased severity. Our model was trained on the full range of GOLD scores, as shown in **Table 1**.

**Table 1:** CODPGene training data.

Gold Score	Qty
0	4387
1	787
2	1926
3	1164
4	606
Uncategorized	1782

Number of CT images per GOLD score.

A total of 13,002 unique breath-hold CT scans, or 6,501 inhale-exhale CT image pairs, from all GOLD scores at the initial time point of the COPDGene imaging study were preprocessed prior to the training phase. Scans were first converted from Hounsfield units, which are calculated based on the attenuation coefficient of the X-ray beam, to density values ranging from 0 to 1 (15). All lung tissues fall within this density range while excluding denser tissue and bone. In order to satisfy computational and memory constraints, the initial full-resolution training CT scans and their corresponding masks were then downsampled with nearest-neighbor interpolation to a resolution of 128x128x128.

### 2.2 SwinUNETR training

**Figure 2:**
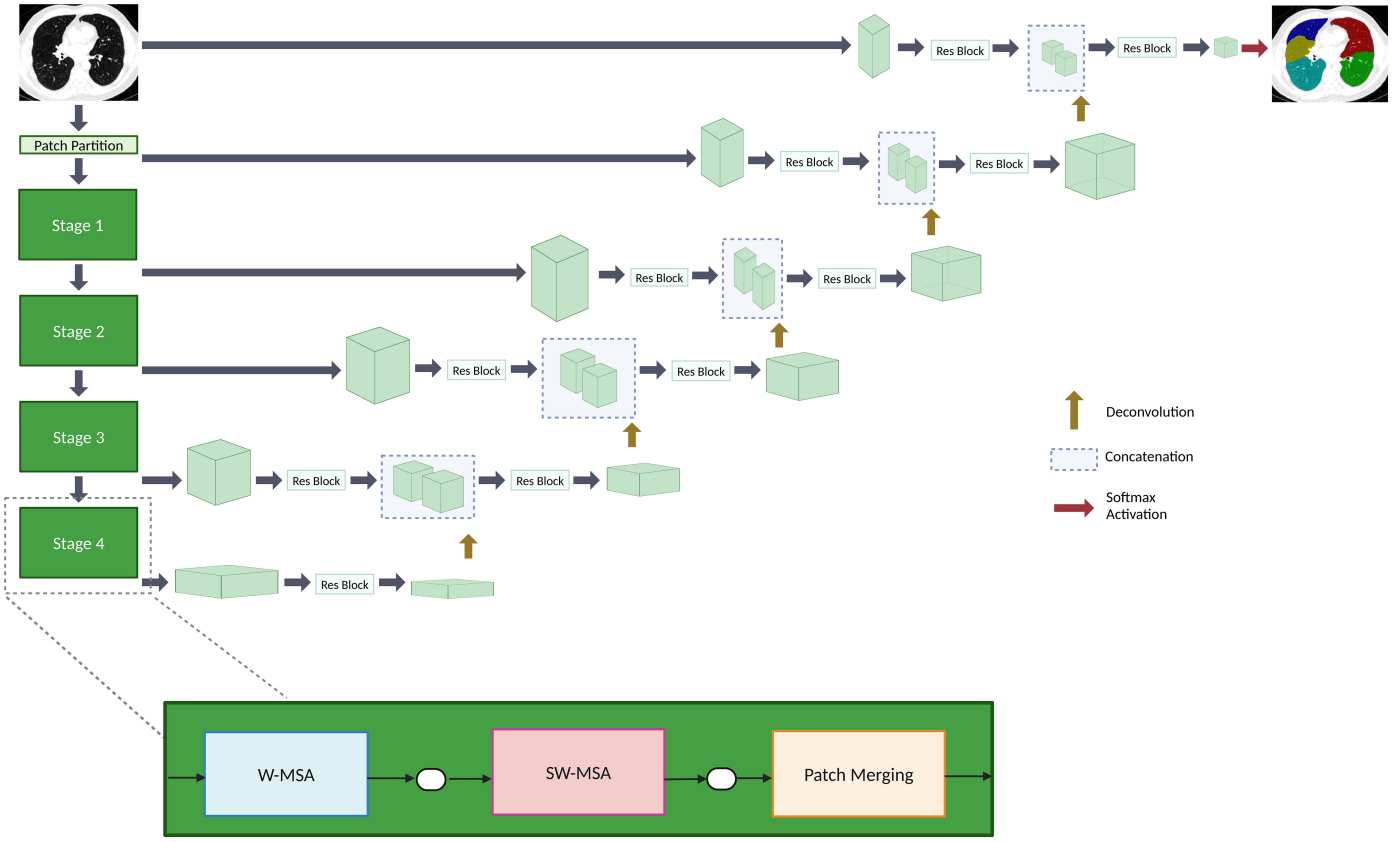
An overview of the SwinUNETR architecture. The encoder contains four stages. Each stage is composed of a window multi-head self-attention (W-MSA) transformer block, a sliding window multi-head self-attention (SW-MSA) transformer block, and a patch merging mechanism. The decoder reshapes output features that are sent to a convolutional block up on the path. The encoder and decoder are connected via skip connections. A softmax activation function outputs the final segmentations.

This paper proposes a lobe segmentation pipeline, TriSwinUNETR, composed of three SwinUNETR networks. The overview of the SwinUNETR architecture is shown in **Figure 2**. The SwinUNETR is a “U-shaped” network composed of a Swin-transformer encoder and a CNN-based decoder with skip connections at each resolution. The encoder begins with a patch partition layer and then proceeds to contain 4 stages comprising 2 transformer blocks each. Each stage contains a window multi-head self-attention transformer mechanism, applied individually within each partition, and a sliding window multi-head self-attention transformer mechanism, applied across different local windows. Patch merging occurs at the end of each stage. Encoded feature representations are concatenated to the decoder input via skip connections at each resolution along the path. In each of the four stages, output features are reshaped and sent to a convolutional residual block. The final segmentation is outputted using a 1x1x1 convolutional layer and softmax activation function. Hyperparameter and optimizer details are included on the - **Supplementary Material** page.

**Figure 3:**
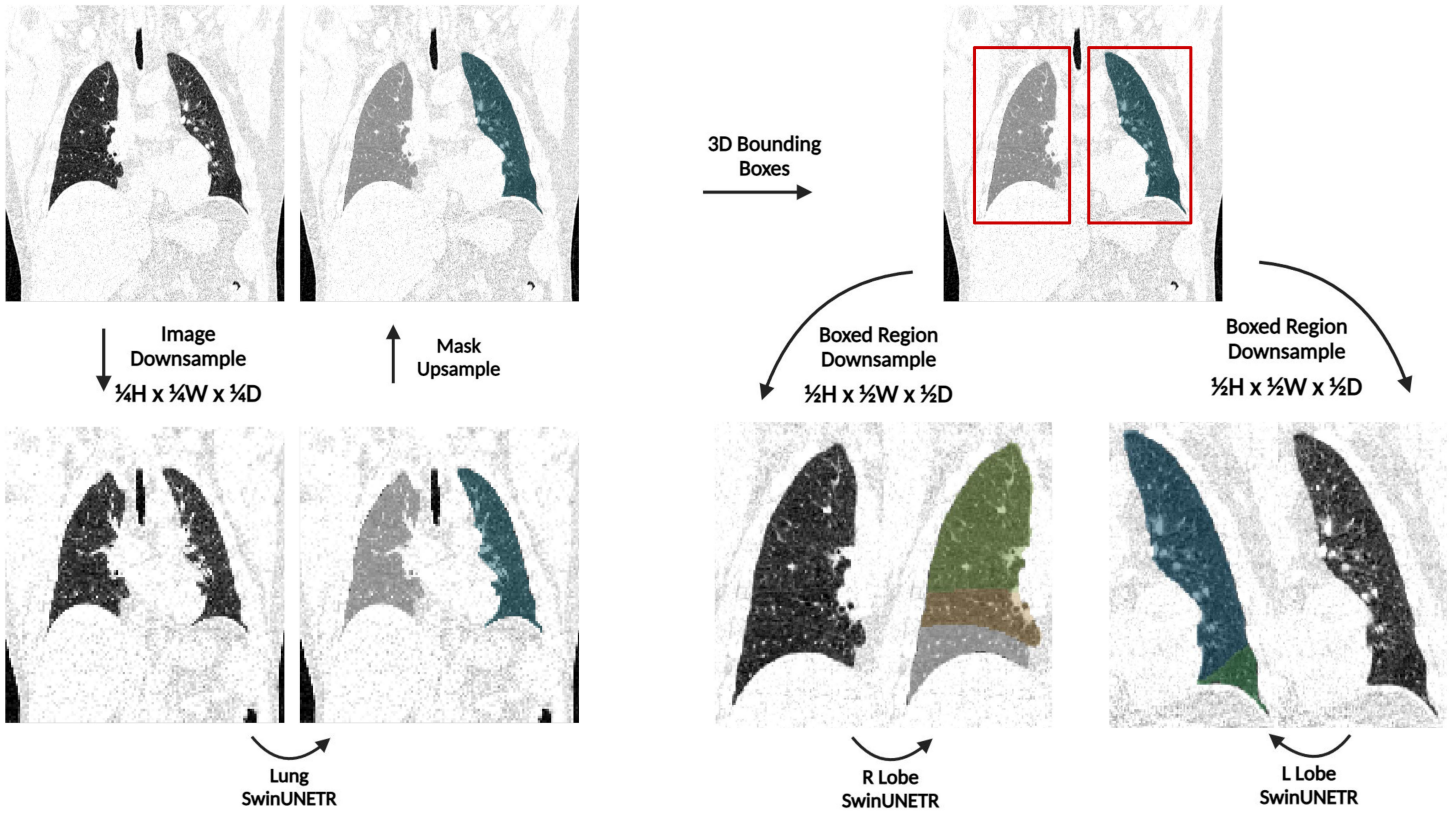
An overview of the TriSwinUNETR pipeline. A full-resolution (512x512x512) CT image is first downsampled to 128x128x128, left and right lung segmentations are acquired then upsampled back to the original image space, bounding boxes for each lung are determined based on the initial segmentations, regions delineated by the bounding boxes are downsampled, then corresponding Right Lobe and Left Lobe SwinUNETR networks output the corresponding lobe segmentations for each lung.

As shown in **Figure 3**, an initial network denoted as Lung SwinUNETR provides right and left lung segmentations on a resolution space of 128x128x128, which are then subsequently utilized to determine bounding boxes for each lung. The bounding boxes acquired from the left and right lung segmentations from the first network are then upsampled and localized back onto the original CT scan, which is at a full resolution of 512x512x512. Each individual lung is then cropped from the original scan, downsampled back to 128x128x128, and provided as input to a dedicated SwinUNETR network that is trained to output the corresponding amount of segmentation classes for lobes in that lung. This strategy only downsamples the lung region instead of the entire CT scan for the task of lobe segmentation, thus preserving a substantial amount of contextual information.

### 2.3 Post-processing

After each model forward pass, the result was dusted with an implementation of a block-based union-find algorithm. All connected components were evaluated with a connectivity of 26 and a threshold of 5000 voxels for the initial segmentation and 3000 voxels for the subsequent segmentations was used for dusting.

### 2.4 Fine-tuning and testing datasets

As shown in **Table 2**, two datasets were used to finetune the L Lobe SwinUNETR and R Lobe SwinUNETR networks. The first fine-tuning step was done on 22 4DCT scans of non-small cell lung cancer patients, with lobe segmentations manually delineated by experts. These scans were obtained as part of a study to incorporate lung function imaging into radiation therapy to preserve function after treatment. Of the 202 inhale and exhale phases from the 101 participants in the study, 22 were randomly selected after validation of image quality (16).

**Table 2:** Summary of datasets used.

Name and Source	Number of CT images	Purpose
COPDGene (14)	13,002	Training Lung, Right Lobe, and Left Lobe SwinUNETR models
4DCT Lung Cancer (18)	22	Fine-tuning Right Lobe and Left Lobe SwinUNETR models
LUNA16 (17)	51	K-fold cross-validation of Right Lobe and Left Lobe SwinUNETR models

The name, source, number of CT images used, and purpose of each dataset in this study are included in this table.

The second dataset used is a subset of the LUNA-16 dataset with lobe segmentations manually created by radiologists. The LUNA-16 dataset was originally a dataset of 888 scans selected from the LIDC-IDRI dataset of lung CT scans with nodules as part of a lung nodule segmentation Grand Challenge in 2016. From this dataset, 51 scans were selected, segmented by radiologists, and presented in (17).

### 2.5 Fine-tuning with 4DCT lung cancer dataset

4DCT non-small cell lung cancer images used in fine-tuning were preprocessed similarly to the COPDGene cases described previously. The process of acquiring bounding boxes for the lungs and using them to crop to each lung on the high-resolution image is the same. However, the two new images and their corresponding lobe masks were not resized to 128^3^; instead, they were resized to the largest possible resolution divisible by 32 with a volume less than 6*128^3^ to fit into memory. This fine-tuning process yielded the best results and was conducted on the right and left networks separately. Hyperparameter and optimizer details are included on the - **Supplementary Material** page.

### 2.6 K-fold cross validation with LUNA16

Following fine-tuning on the cancer dataset, K-fold cross-validation was performed on a random subset of 51 LUNA16 lobe segmentation cases, as previously described. The original LUNA16 scans were preprocessed in the same manner as the COPDGene dataset. The 51 cases were divided into k = 5 folds of 10 or 11 cases each. The optimizer and hyperparameters used were identical to the previous fine-tuning step. The accuracy of segmentations was calculated using Dice percent score.

### 2.7 CT-ventilation calculation

**Figure 4:**
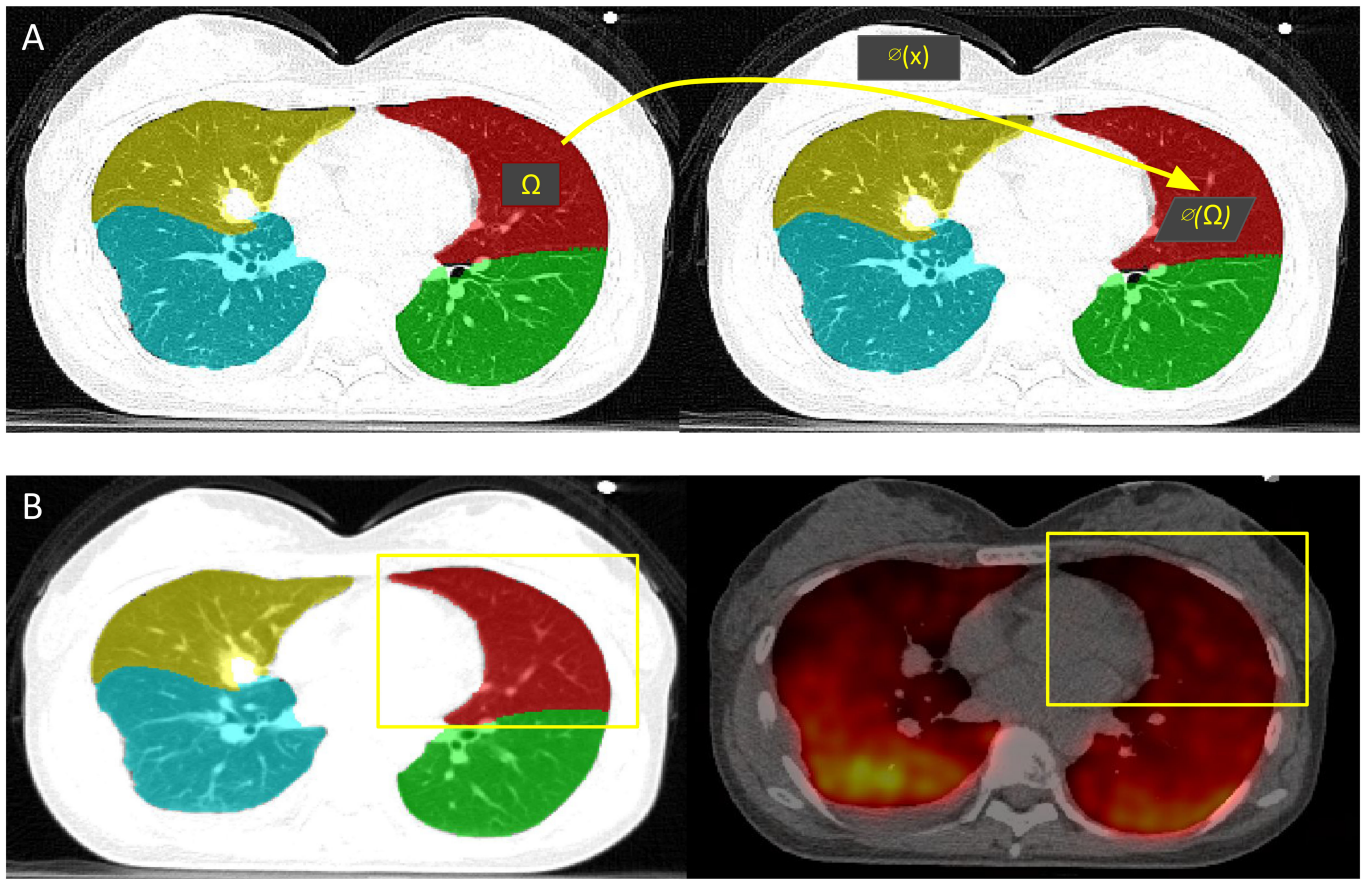
CTVI calculation and validation. **(A)** CTVI is calculated based on the change in volume between the inhale (right) and exhale (left) segmentations, where ventilation(Ω) = |1 - vol(∅(Ω))/vol(Ω)|. **(B)** The ground-truth PET-Galligas (right) is acquired by isolating the number of counts per lobe based on the TriSwinUNETR lobe segmentation from the PET-CT scan (left).

Using the trained and fine-tuned AI model previously described, lobe segmentations were acquired from the inhale and exhale phases of the 4DCT of 19 lung cancer patients from a publicly available dataset (18). As shown in **Figure 4**, CTVI calculation for each lobe was acquired by determining the breathing-induced lobar volume change using AI-defined lobe segmentation volumes, without DIR. The CTVI was calculated for each individual patient as the percent change in lobar volumetric segmentations between the full inhale and full exhale phases of the breathing cycle. Each patient had five CTVI values, one for each main lobar region.

Resulting lobar CTVI values were validated with positron emission tomography (PET)-Galligas ventilation imaging for each lung cancer patient, which was acquired from the same publicly available dataset (18). As shown in **Figure 4**, the PET-Galligas ventilation was acquired for each patient’s lobe (LUL, LLL, RUL, RML, RLL) based on the number of counts or detected photon events recorded by the PET scanner. Each of the five PET-Galligas ventilation values was compared against the CTVI value calculated for that specific lobe. The spatial Spearman correlation between TriSwinUNETR lobe ventilation and ground-truth PET-Galligas ventilation was calculated for each patient. A Spearman correlation value of at least 0.5 suggests a moderately strong correlation between AI-based ventilation and the ground truth. It is important to note that the PET imaging itself, as shown in **Figure 4**, could have been a source of error since the radioactive material can be seen going beyond the extremities of the lungs. Since the calculations were isolated to lobes only, the radioactive material outside of the lungs was not considered.

To determine whether the proposed volume-change approach may improve CTVI calculations compared to DIR-based methods, the CTVIs for the 19 lung cancer cases were generated using both methods. The chosen DIR-based method to use was the integral formulation of the Jacobian (IJF), which aims to estimate the apparent voxel volume changes within an inhale/exhale CT image pair (19). The Spatial Spearman correlation between IJF ventilation and ground-truth PET-Galligas was generated for each patient.

## 3 Results

### 3.1 Dice comparison for lobe segmentation

**Table 3** shows the lobe segmentation results of previous high-performing model architectures. Our method attained a mean Dice percent score of 93.75 ± 1.81% on the LUNA16 cases, with RUL at 93.49 ± 2.76%, RML at 85.78 ± 5.61%, RLL at 95.65 ± 0.69%, LUL at 97.12 ± 0.17%, and LLL at 96.58 ± 0.42%. 4.08M working parameters were used in the Lung SwinUNETR, and 15.7M working parameters were used in the L Lobe SwinUNETR and R Lobe SwinUNETR each. TriSwinUNETR contains 35.48M working parameters in total.

**Table 3:** Dice percent score comparison.

Model	RUL Dice %	RML Dice %	RLL Dice %	LUL Dice %	LLL Dice %	Mean Dice %	Params (M)
UNETR (11)	92.47	83.56	93.15	93.51	92.84	91.11	101.96
UNet++ (20)	92.20	82.26	94.08	95.89	95.53	92.00	N/A
AttentionUnet (21)	93.01	82.91	94.24	95.32	94.89	92.07	23.63
SCLMnet (22)	92.81	78.28	95.02	97.68	97.05	92.17	87.31
**TriSwinUNETR**	**93.50**	**85.82**	**95.65**	97.16	96.61	**93.75**	35.48

Dice percent scores for RUL, RML, RLL, LUL, LLL, and mean Dice percent score are given for each model. Model parameters (in millions) are also included for each model. Bolded values indicate tasks where TriSwinUNETR outperforms existing architectures.

TriSwinUNETR Dice percent scores included on **Table 3** are the average across the five folds from our K-fold cross-validation. **Table 4** shows the results for the K-fold cross validation of 51 LUNA16 cases using TriSwinUNETR network.

**Table 4:** Dice percent scores per fold.

Fold	RUL Dice %	RML Dice %	RLL Dice %	LUL Dice %	LLL Dice %	Mean Dice %
1	93.38	84.80	94.73	97.09	96.32	93.27
2	96.22	92.09	95.91	97.50	97.33	95.81
3	93.09	83.30	96.15	97.13	96.10	93.15
4	88.65	77.14	94.97	96.98	96.75	90.90
5	96.14	91.75	96.51	97.13	96.54	95.61

Dice percent scores for RUL, RML, RLL, LUL, LLL, and mean Dice percent score are given for each fold.

**Figure 5:**
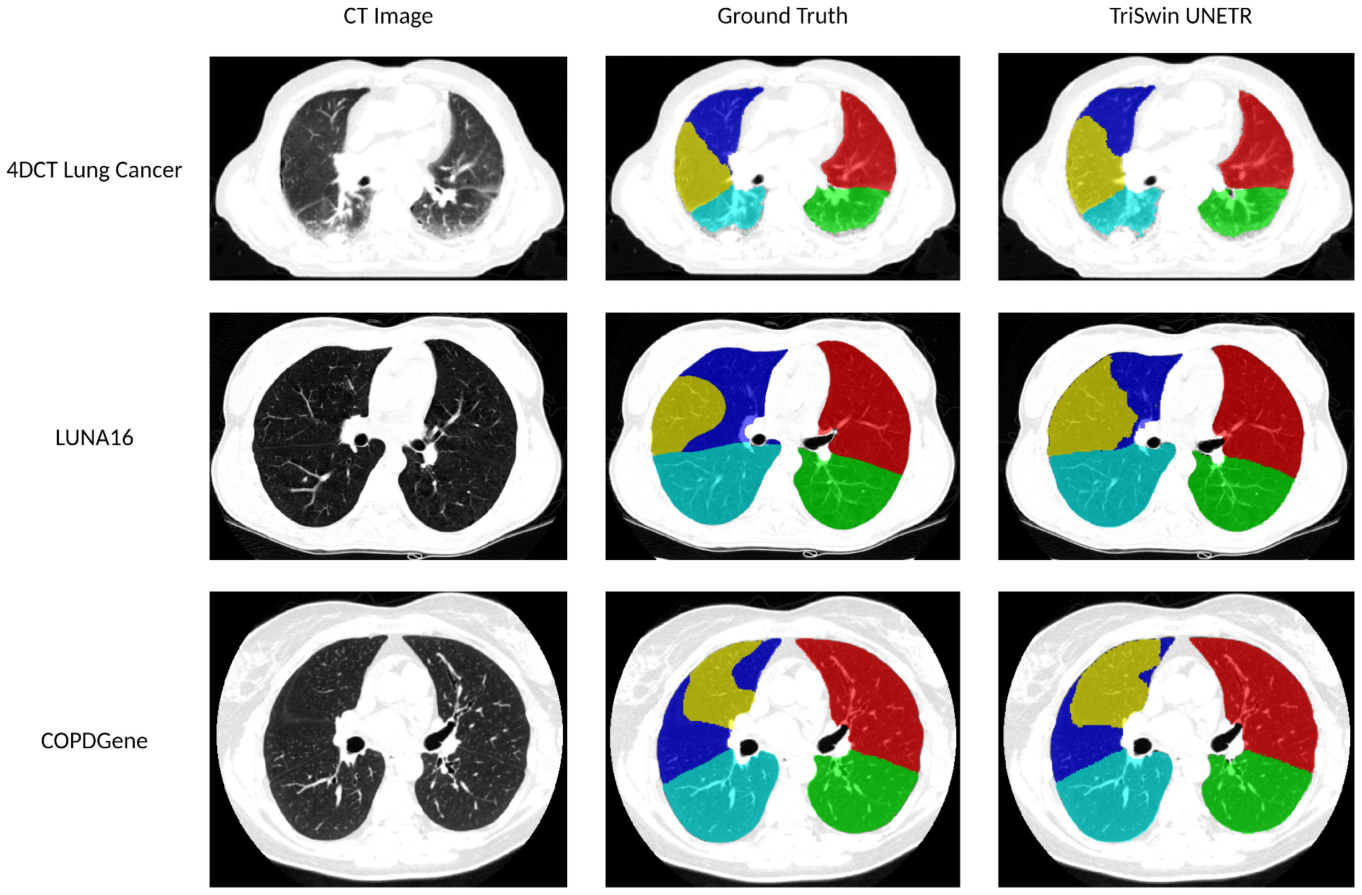
Lobar segmentations comparison. The 4DCT Lung Cancer test case has an average percent Dice score of 92.61 (LUL: 96.77 LLL: 89.69 RUL: 96.26 RML: 93.99 RLL: 86.36). The LUNA16 test case has an average percent Dice score of 96.05 (LUL: 97.45 LLL: 97.69 RUL: 95.16 RML: 92.35 RLL: 97.61). The COPDGene test case has an average percent Dice score of 96.62 (LUL: 97.83 LLL: 97.28 RUL: 97.20 RML: 94.09 RLL: 96.69).

Representative test cases from the three datasets used to train and finetune the model were selected, and their corresponding lobe segmentations and ground truth are shown in **Figure 5**.

### 3.2 Spearman correlation for CT-ventilation

**Table 5** shows the Spearman correlation coefficients between the CTVI and the PET-Galligas for each of the 19 lung cancer patients. The correlation was calculated between the CTVI and PET-Galligas count for all five lobes (LUL, LLL, RUL, RML, RLL) per patient. The median Spearman correlation coefficient was 0.9 across 19 cases, with 13 cases exhibiting correlations of at least 0.5, indicating moderately strong agreement between CTVI and PET-Galligas ventilation.

**Table 5:** Spearman correlation coefficients of proposed method’s CTVI vs. IJF CTVI.

Patient ID	1	2	3	4	5	6	8	9	10	11	12	13	14	15	16	17	18	19	20
**Proposed Method**	**0.9**	0.2	-1	**1**	**0.6**	**0.9**	**0.9**	**0.9**	**0.6**	**0.9**	**0.9**	0	**0.9**	0.3	0.2	**0.5**	**0.9**	-1	**1**
**DIR-Based IJF**	**0.54**	**0.52**	0.39	0.45	**0.56**	0.27	0.48	**0.59**	**0.64**	**0.71**	0.27	0.17	**0.54**	0.42	0.33	0.35	**0.56**	0.41	0.41

Patient ID is the number describing each anonymous patient. Spearman coefficient was calculated from the correlations of the CTVI and PET-Galligas for all five lobes per patient. Bolded numbers represent successful cases (correlation ≥ 0.5). Patient 7’s PET-Galligas ventilation could not be calculated as it lacks a necessary CT image.

**Figure 6:**
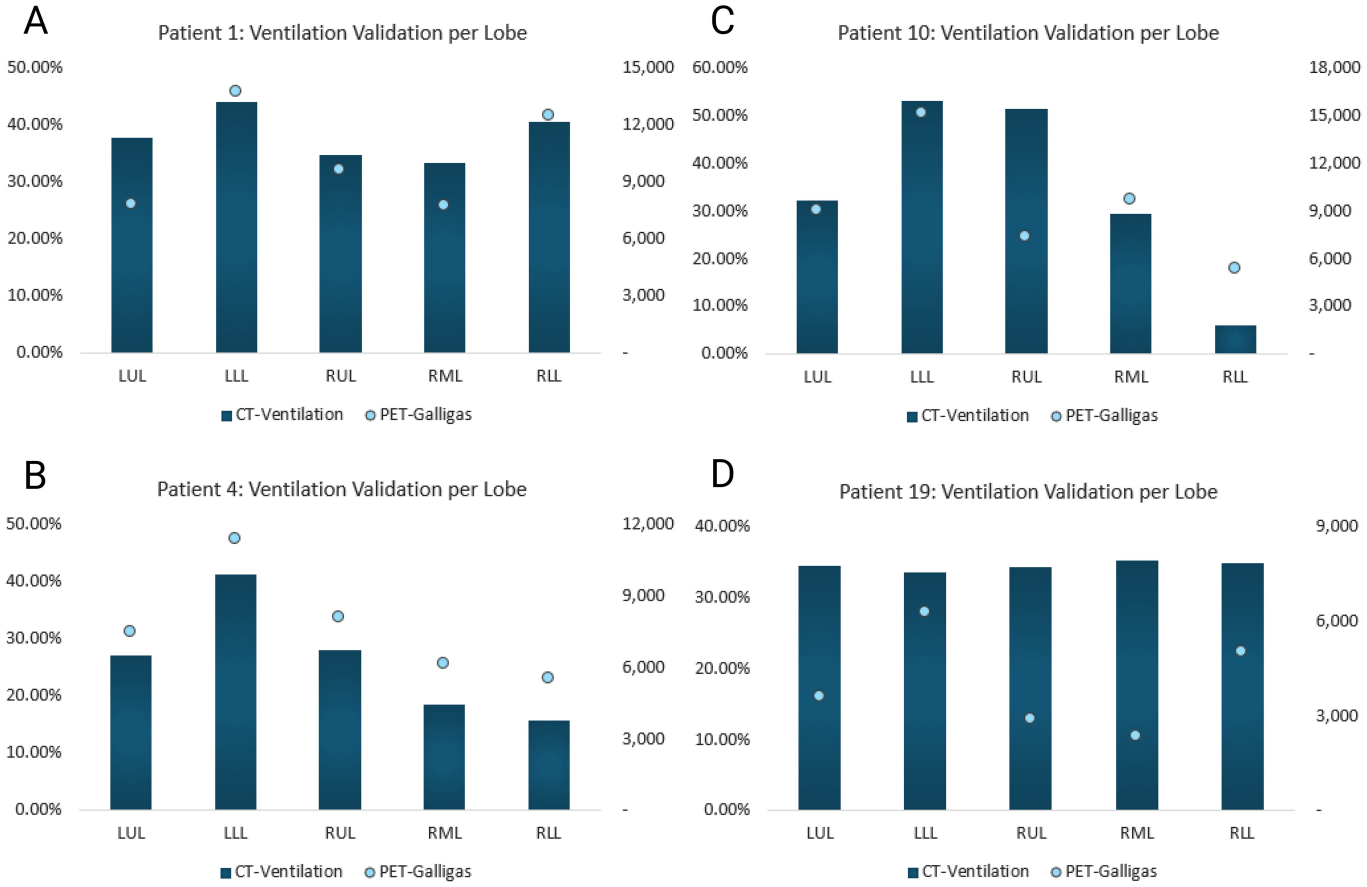
Proposed method’s CTVI vs. PET-Galligas counts per lobe. CTVI is calculated in percent and PET-Galligas ventilation is calculated by the number of counts. The graphs included serve to visualize strong vs. weak Spearman correlations. **(A)** Patient 1’s calculated Spearman correlation = 0.9, **(B)** Patient 4’s calculated Spearman correlation = 1, **(C)** Patient 10’s calculated Spearman correlation = 0.6, **(D)** Patient 19’s calculated Spearman correlation = -1.

**Figure 6** compares the proposed method’s CTVI values and the number of PET-counts per lobar region for four patients. As shown, higher percent ventilation values should correspond to higher numbers of counts detected by the PET-scanner in order to result in a strong Spearman correlation. Refer to the - **Supplementary Material** page for all patients’ data.

## 4 Discussion

### 4.1 Lobe segmentation task

Lung cancer is the leading cause of cancer death in the United States, causing more deaths in 2020 than breast, colorectal, and prostate cancers combined (23). However, advancements in the understanding of tumor biology, development of targeted therapies, and introduction of low-dose computed tomography (LDCT) for lung cancer screening have increased survival rates (23). Annual cancer screening using LDCT is an integral step in detecting cancer at its earlier stages, and lobe segmentation is a necessary part of the process. Computer-assisted diagnosis (CAD) methods aid radiologists in early lung nodule detection, but to do so, automatic segmentation of pulmonary lobes must be completed to eliminate other confounding structures such as the heart, the thoracic wall, abdominal organs, and the vertebrae (24). Although current automatic lobe segmentation models exist, previous methods may fail to train on different lung conditions and disease states in addition to struggling to downsample images without losing substantial contextual information, as previously discussed (6, 7). For these reasons we propose a novel CT lobe segmentation pipeline (TriSwinUNETR), which employs transfer learning to ensure generalizability to multiple datasets and disease states and breaks down segmentation tasks to prevent loss of contextual information caused by image downsampling.

In the first part of this study, we used K-fold cross-validation applied to the LUNA16 dataset to compare against previously published results. Although previous works do not mention the process of K-fold cross-validation nor a clear method for calculating standard deviation of Dice scores, we have chosen to perform 5-fold validation to ensure that LUNA16 test results are robust and reproducible. The proposed TriSwinUNETR achieves a mean Dice score of 93.75%, surpassing the mean Dice percent accuracy reported by the current state-of-the-art model, SCLMnet (22). In particular, TriSwinUNETR outperforms pre-existing architectures on the segmentation of lobes in the right lung.

As shown in **Figure 5**, our pipeline performs well in comparison to ground truth manual segmentations. Since we downsample a smaller portion of the image instead of the entire CT scan, a significant amount of information is preserved on the lower-resolution image, thus improving the accuracy of our results. In addition, fine-tuning on higher-resolution lung cancer images preserved higher-quality contextual information that could have been useful in the network’s learning process. Due to their vast number of parameters as well as the complexity of architecture, transformer-based models require a significant amount of images to learn (25). Therefore, in addition to preserving as much of the image resolution as possible, transfer learning also increased the accuracy of our results. Training the models first on the COPDGene dataset allowed the architecture to familiarize itself with lung anatomy and the segmentation task. However, fine-tuning on the lung cancer dataset prior to doing K-fold cross-validation on LUNA16 allowed us to ensure that the model would be exposed to different types of scanners and lung conditions.

A limitation of our study is the quality of the training data itself. As previously stated, transformer-based models require a substantial amount of images to be optimized, but manual lobe segmentation is a time-consuming task. Therefore, we trained TriSwinUNETR on the larger COPDGene dataset, despite its automated segmentations that are prone to errors. This bias could have influenced the training of our model. However, in order to minimize errors caused by such bias, we have fine-tuned the Left and Right SwinUNETR networks with 62/63 images (22 lung cancer and 40/41 lung nodule CTs). In addition, our pipeline, along with other existing methods, struggles to segment cases where the patient has undergone a lobectomy due to the lack of available lobe segmentations of cases involving lobectomies. Therefore, future directions for this work include expanding the datasets and disease states that we train our models on, in addition to developing an even more effective method to preserve image resolution prior to the segmentation task.

### 4.2 CT-ventilation task

Given the high mortality of lung cancer in the United States, radiotherapy (RT) has been undergoing several technological innovations in recent years (26). One specific type of RT, functional RT, allows irradiation of tumors with high doses, while sparring healthy lung tissue. In order to make use of this advancement, however, functional image information is necessary to determine high-functioning lung areas in addition to applying personalized dose prescriptions for patients (26). CTVI is a functional metric that has been positively correlated with high-functioning areas of the lung, as previously discussed (4). Deformable image registration (DIR) is a core process in radiotherapy treatment planning and in calculating CTVI. However, DIR is a process that requires caution, as previously stated, as it is a process that is highly subject to variation in algorithm and user input (27). It has also been observed that DIR may return results that are physically implausible (27). Therefore, in the second part of this study, we propose the use of our TriSwinUNETR network to calculate CTVI without the use of DIR.

The Spearman correlation coefficients reveal that when using the proposed lobe volume change method, 13 out of 19 lung cancer cases (~68%) yielded successful CTVI results when compared to the ground-truth PET-Galligas per lobe. On the other hand, when using the DIR-based IJF method, only 8 out of 19 lung cancer cases (~42%) yielded successful CTVI results compared to ground truth. These results indicate the potential benefit of implementing a lobe volume change approach to calculate CTVI instead of solely relying on DIR. Since the lobe segmentation pipeline has been proven over 93% accurate on LUNA16 cases and has been fine-tuned on lung cancer cases, the model is well-prepared to segment lobes from the lung cancer 4DCT test set. Possible reasons for the proposed model’s Spearman correlation coefficients < 0.5 include patients who have had a lobectomy, which as previously discussed could be challenging for the model to segment. In addition, the 4DCT lung cancer cases have an image resolution of 512 x 512 x ~170; the lower image resolution on the z-axis could make it more challenging for the AI model to identify fissures in certain patients. Lastly, most of the unsuccessful cases have abnormal PET-Galligas, which are either incorrectly cropped or missing to fill a sublobar region. Refer to the - **Supplementary Material** page to see figures of patients’ with Spearman correlation < 0.5.

Possible future directions for this work include combining the proposed CTVI method with an iterative DIR method. While CTVI calculations per lobe provide interpretable ventilation results and can be verified by referencing lobe segmentation outputs, iterable DIR-methods allows for every voxel to be registered from inhale to exhale phase instead of comparing the volume change of a lobar region. Therefore, a combination of iterative DIR limited to a specific lobar region outputted by the AI model, or DIR-ventilation results cross-referenced to the AI-outputted lobe ventilation results could yield a mathematically stable and accurate CTVI method.

### 4.3 Ethical considerations

Our proposed method of calculating CTVI directly from lobe segmentations has the potential to be implemented in the clinic. In the United States, 4DCT imaging is a standard aspect of radiotherapy treatment planning for patients with lung cancer (28). Therefore, acquiring lobe segmentations directly from patients’ CT images and performing CTVI calculations in local hospital machines would not disrupt the clinical workflow or risk patient data leaking. With the appropriate quality assurance procedures already in place at radiation oncology clinics, AI-defined lobe segmentations and their corresponding ventilation values may be inspected by medical physicists and radiation oncologists prior to implementation. The benefits of CTVI implementation in the clinic have been shown in a 2022 study. It was proven that CTVI as a functional imaging metric for functional avoidance radiotherapy planning reduced pneumonitis rates in lung cancer patients, thus proving CTVI’s potential for clinical implementation (28).

## 5 Conclusion

In this work, we proposed a novel implementation of state-of-the-art segmentation architecture for automated CT lobe segmentation and made it publicly available to the scientific community. We utilized a TriSwinUNETR composed of three SwinUNETR networks for three distinct segmentation tasks: left and right lung segmentation, right lobes segmentation, and left lobes segmentation. Our proposed method, trained on a section of the COPDGene dataset and fine-tuned on manual lobe segmentations, includes minimal preprocessing and postprocessing. Dice score comparison on a subsection of the LUNA16 dataset showed that our proposed method outperforms currently proposed state-of-the-art methods. Using the proposed TriSwinUNETR AI-defined lobe volumes from a 4DCT lung cancer dataset, we have calculated the per-patient CTVI value for each lobe. Spatial Spearman correlation between TriSwinUNETR lobe ventilation and ground-truth PET-Galligas ventilation indicates strong agreement, thus possibly revealing a DIR-free alternative for calculating CTVI with the use of an AI-based lobe segmentation model. Future directions for this work include developing a more effective method to preserve image resolution prior to the segmentation task, expanding training datasets to include more disease states, and possibly stabilizing the mathematical uncertainties of DIR calculations with the proposed AI-based CTVI method.

## Data Availability

Publicly available datasets were analyzed in this study. This data can be found here: https://luna16.grand-challenge.org/, https://copdgene.org/, https://www.cancerimagingarchive.net/collection/ct-vs-pet-ventilation-imaging/. Trained model weights for this study can be found in the TriSwinUNETR repository at https://github.com/DMIC-Lab/segmentation-pipeline.
