# Supplementary material for: TriSwinUNETR lobe segmentation model for computing DIR-free CT-ventilation
Source: Front Oncol. 2025 Feb 17;15:1475133. doi: 10.3389/fonc.2025.1475133 (PMC11872890; doi:10.3389/fonc.2025.1475133)
Supplement: Supplementary file 1 [file DataSheet1.docx]

Supplementary Material

# Details of Methods and Materials

## SwinUNETR Training Parameters

All models were instantiated as Pytorch modules with open-source implementations from the Medical Open Network for Artificial Intelligence (MONAI). All models were trained with a batch size of 6, image size of 128^3^, dice loss, and an AdamW optimizer. The initial network has a hidden feature size of 12, while the lobe segmentation networks have a hidden feature size of 24. The initial network and lobe segmentation networks were trained with a learning rate of 1e-3 and 1e-4, respectively.

## 4D-CT Lung Cancer Dataset Fine-Tuning Parameters

Fine-tuning the left and right network with the 4D-CT lung cancer dataset was done with an AdamW optimizer with a learning rate of 1e-6, batch size of 1, and a Dice Loss function. Lung tumors present in the CT images were labeled as the background class.

# Detailed Results of Spearman Correlation for CT-Ventilation

## Volume-Based CT-Ventilation vs. PET-Counts Comparison per Patient

|  | **LUL** | **LLL** | **RUL** | **RML** | **RLL** |  |
| --- | --- | --- | --- | --- | --- | --- |
| **Patient 1** | 37.81% | 44.22% | 34.70% | 33.48% | 40.63% |  |
|  | 7,847 | 13,737 | 9,622 | 7,756 | 12,503 |  |
| **Patient 2** | 7.76% | 4.36% | 1.41% | 6.29% | 3.48% |  |
|  | 7,033 | 8,730 | 4,045 | 6,193 | 8,422 |  |
| **Patient 3** | 0.97% | 1.41% | 0.68% | 2.01% | 5.19% |  |
|  | 5,982 | 6,180 | 5,674 | 4,226 | 5,598 |  |
| **Patient 4** | 27.10% | 41.35% | 28.05% | 18.43% | 15.74% |  |
|  | 7,477 | 11,379 | 8,113 | 6,152 | 5,533 |  |
| **Patient 5** | 25.19% | 31.81% | 10.38% | 25.73% | 25.18% |  |
|  | 8,518 | 10,706 | 983 | 1,375 | 9,351 |  |
| **Patient 6** | 23.89% | 37.67% | 19.46% | 17.69% | 37.25% |  |
|  | 3,105 | 5,663 | 2,194 | 3,044 | 4,865 |  |
| **Patient 7** | N/A | N/A | N/A | N/A | N/A |  |
|  | N/A | N/A | N/A | N/A | N/A |  |
| **Patient 8** | 12.21% | 34.55% | 19.55% | 14.13% | 32.55% |  |
|  | 419 | 1,086 | 576 | 715 | 952 |  |
| **Patient 9** | 6.25% | 43.13% | 12.46% | 3.30% | 46.79% |  |
|  | 7,600 | 14,369 | 8,956 | 4,793 | 14,330 |  |
| **Patient 10** | 32.16% | 53.34% | 51.56% | 29.56% | 5.93% |  |
|  | 9,094 | 15,135 | 7,405 | 9,766 | 5,353 |  |
| **Patient 11** | 34.09% | 52.40% | 26.71% | 14.74% | 39.53% |  |
|  | 591 | 767 | 327 | 303 | 506 |  |
| **Patient 12** | 26.63% | 23.44% | 28.96% | 22.48% | 34.83% |  |
|  | 4,461 | 3,631 | 4,294 | 3,306 | 4,952 |  |
| **Patient 13** | 10.90% | 17.28% | 8.72% | 11.18% | 13.50% |  |
|  | 1,980 | 2,591 | 2,397 | 989 | 327 |  |
| **Patient 14** | 40.14% | 41.07% | 38.39% | 27.49% | 49.63% |  |
|  | 2,577 | 3,332 | 1,898 | 1,671 | 3,256 |  |
| **Patient 15** | 12.23% | 16.66% | 10.36% | 87.76% | 12.04% |  |
|  | 5,075 | 4,985 | 800 | 1,231 | 1,737 |  |
| **Patient 16** | 30.96% | 40.12% | 31.12% | 36.19% | 38.87% |  |
|  | 10,066 | 12,508 | 12,899 | 9,114 | 11,930 |  |
| **Patient 17** | 20.54% | 27.97% | 25.65% | 7.39% | 29.23% |  |
|  | 5,741 | 12,354 | 5,589 | 5,974 | 9,860 |  |
| **Patient 18** | 34.20% | 53.14% | 36.86% | 27.46% | 50.50% |  |
|  | 4,131 | 5,764 | 4,043 | 2,951 | 4,560 |  |
| **Patient 19** | 34.52% | 33.53% | 34.29% | 35.26% | 34.88% |  |
|  | 3,620 | 6,293 | 2,893 | 2,341 | 5,019 |  |
| **Patient 20** | 23.57% | 34.20% | 22.43% | 16.79% | 37.58% |  |
|  | 2,574 | 3,773 | 2,318 | 1,655 | 4,134 |  |

**Supplementary Figure 1.** CT-ventilation and ground-truth PET Galligas values of lung cancer patients. The CT-ventilation percentage and the PET-Galligas number of counts per lobe for each patient are included in the table.

## PET-Galligas and Lobar Segmentations for Failed Cases


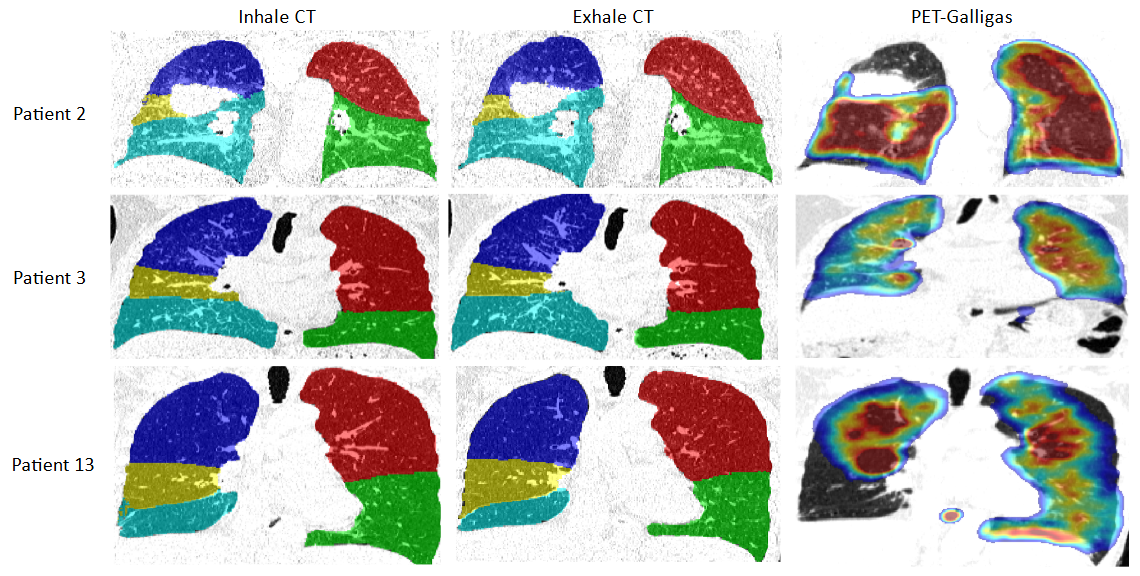


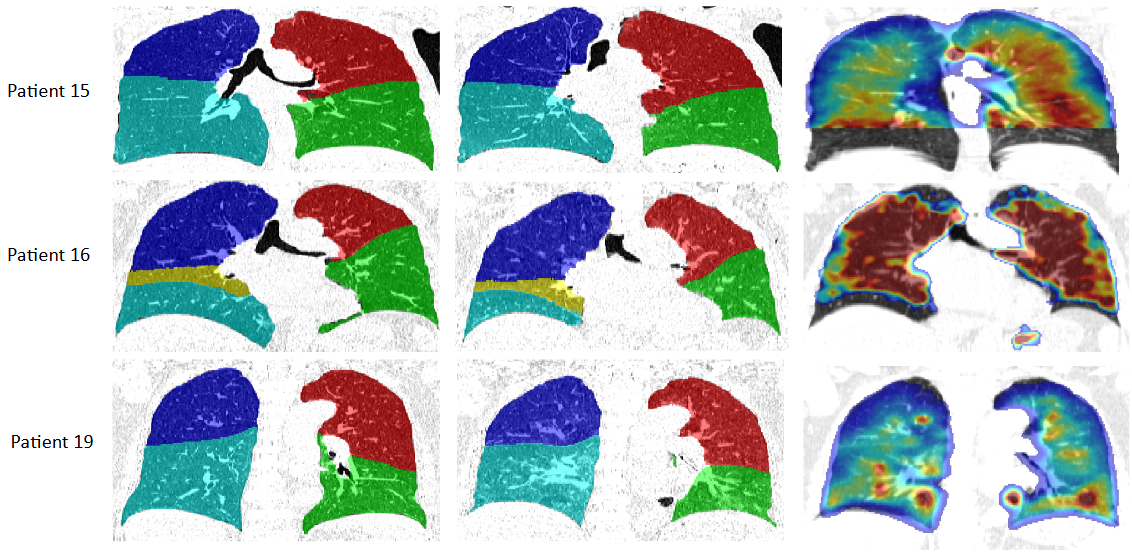


**Supplementary Figure 2.** Ventilation imaging of cases with low Spearman correlation. A representative slice is shown from the inhale CT, exhale CT, and PET-Galligas image from the six failed cases (Spearman correlation < 0.5) in Section 3.2.
